# Supplementary material for: The Effects of Concurrent Training Combined with Low-Carbohydrate High-Fat Ketogenic Diet on Body Composition and Aerobic Performance: A Systematic Review and Meta-Analysis
Source: Int J Environ Res Public Health. 2022 Sep 14;19(18):11542. doi: 10.3390/ijerph191811542 (PMC9517144; doi:10.3390/ijerph191811542)

Supplementary Materials

Supplementary Methods S1: Detailed Search Strategy 2

Supplementary Figure S1: Funnel plot of the effect of CT combined with LCHF on lean mass compared to controls. 3

Supplementary Figure S2A: Forest plot of the effect of CT combined with LCHF on body fat percentage in recreationally trained or professionally trained participants. 4

Supplementary Figure S2B: Forest plot of the effect of intervention duration (≤6 weeks or > 6 weeks) on body fat percentage. 5

Supplementary Figure S3: Funnel plot of the effect of CT combined with LCHF on body fat percentage to controls. 6

Supplementary Figure S4A: Forest plot of the effect of CT combined with LCHF on body mass in recreationally trained or professionally trained participants. 7

Supplementary Figure S4B: Forest plot of the effect of intervention duration (≤6 weeks or > 6 weeks) on body mass. 8

Supplementary Figure S5: Funnel plot of the effect of CT combined with LCHF on body mass. 9

Supplementary Figure S6: Funnel plot of the effect of CT combined with LCHF on VO2max. 10

Supplementary Figure S7A: Forest plot of the effect of CT combined with LCHF on aerobic performance in recreationally trained or professionally trained participants. 11

Supplementary Figure S7B: Forest plot of the effect of intervention duration (≤6 weeks or > 6 weeks) on aerobic performance. 12

Supplementary Figure S8: Funnel plot of the effect of CT combined with LCHF on aerobic performance. 13

Supplementary Methods S1: Detailed Search Strategy

Search Strategy

[‘Diet, Carbohydrate Restricted’ or ‘Diet, Low Carbohydrate’ or ‘Carbohydrate Diet, Low’ or ‘Carbohydrate Diets, Low’ or ‘Diets, Low Carbohydrate’ or ‘Low Carbohydrate Diets’ or ‘Carbohydrate-Restricted Diet’ or ‘Carbohydrate Restricted Diet’ or ‘Carbohydrate-Restricted Diets’ or ‘Diets, Carbohydrate-Restricted’ or ‘Low-Carbohydrate Diet’ or ‘Diet, Low-Carbohydrate’ or ‘Diets, Low-Carbohydrate’ or ‘Low Carbohydrate Diet’ or ‘Low-Carbohydrate Diets’ or ‘Ketogenic Diet’ or ‘Diets, Ketogenic’ or ‘Ketogenic Diets’ or ‘Diets, High-Fat’ or ‘High-Fat Diet’ or ‘High-Fat Diets’ or ‘Diet, High Fat’ or ‘Diets, High Fat’ or ‘High Fat Diet’ or ‘High Fat Diets’] AND [‘Exercises’ or ‘Physical Activity’ or ‘Activities, Physical’ or ‘Activity, Physical’ or ‘Physical Activities’ or ‘Exercise, Physical’ or ‘Exercises, Physical’ or ‘Physical Exercise’ or ‘Physical Exercises’ or ‘Physical Exercises’ or ‘Acute Exercise’ or ‘Acute Exercises’ or ‘Exercise, Acute’ or ‘Exercises, Acute’ or ‘Exercise, Isometric’ or ‘Exercises, Isometric’ or ‘Isometric Exercises’ or ‘Isometric Exercise’ or ‘Exercise, Aerobic’ or ‘Aerobic Exercise’ or ‘Aerobic Exercises’ or ‘Exercises, Aerobic’ or ‘Exercise Training’ or ‘Exercise Trainings’ or ‘Training, Exercise’ or ‘Trainings, Exercise’ or ‘Training, Endurance’ or ‘High Intensity Interval Training’ or ‘High-Intensity Interval Trainings’ or ‘Interval Training, High-Intensity’ or ‘Interval Trainings, High-Intensity’ or ‘Training, High-Intensity Interval’ or ‘Trainings, High-Intensity Interval’ or ‘High-Intensity Intermittent Exercise’ or ‘Exercise, High-Intensity Intermittent’ or ‘Exercises, High-Intensity Intermittent’ or ‘High-Intensity Intermittent Exercises’ or ‘Sprint Interval Training’ or ‘Sprint Interval Trainings’ or ‘Training, Resistance’ or ‘Strength Training’ or ‘Training, Strength’ or ‘Weight-Lifting Strengthening Program’ or ‘Strengthening Program, Weight-Lifting’ or ‘Strengthening Programs, Weight-Lifting’ or ‘Weight Lifting Strengthening Program’ or ‘Weight-Lifting Strengthening Programs’ or ‘Weight-Lifting Exercise Program’ or ‘Exercise Program, Weight-Lifting’ or ‘Exercise Programs, Weight-Lifting’ or ‘Weight Lifting Exercise Program’ or ‘Weight-Lifting Exercise Programs’ or ‘Weight-Bearing Strengthening Program’ or ‘Strengthening Program, Weight-Bearing’ or ‘Strengthening Programs, Weight-Bearing’ or ‘Weight Bearing Strengthening Program’ or ‘Weight-Bearing Strengthening Programs’ or ‘Weight-Bearing Exercise Program’ or ‘Exercise Program, Weight-Bearing’ or ‘Exercise Programs, Weight-Bearing’ or ‘Weight Bearing Exercise Program’ or ‘Weight-Bearing Exercise Programs’ or ‘Concurrent training’ or ‘cross fit’]

Supplementary Figure S1: Funnel plot of the effect of CT combined with LCHF on lean mass compared to controls.

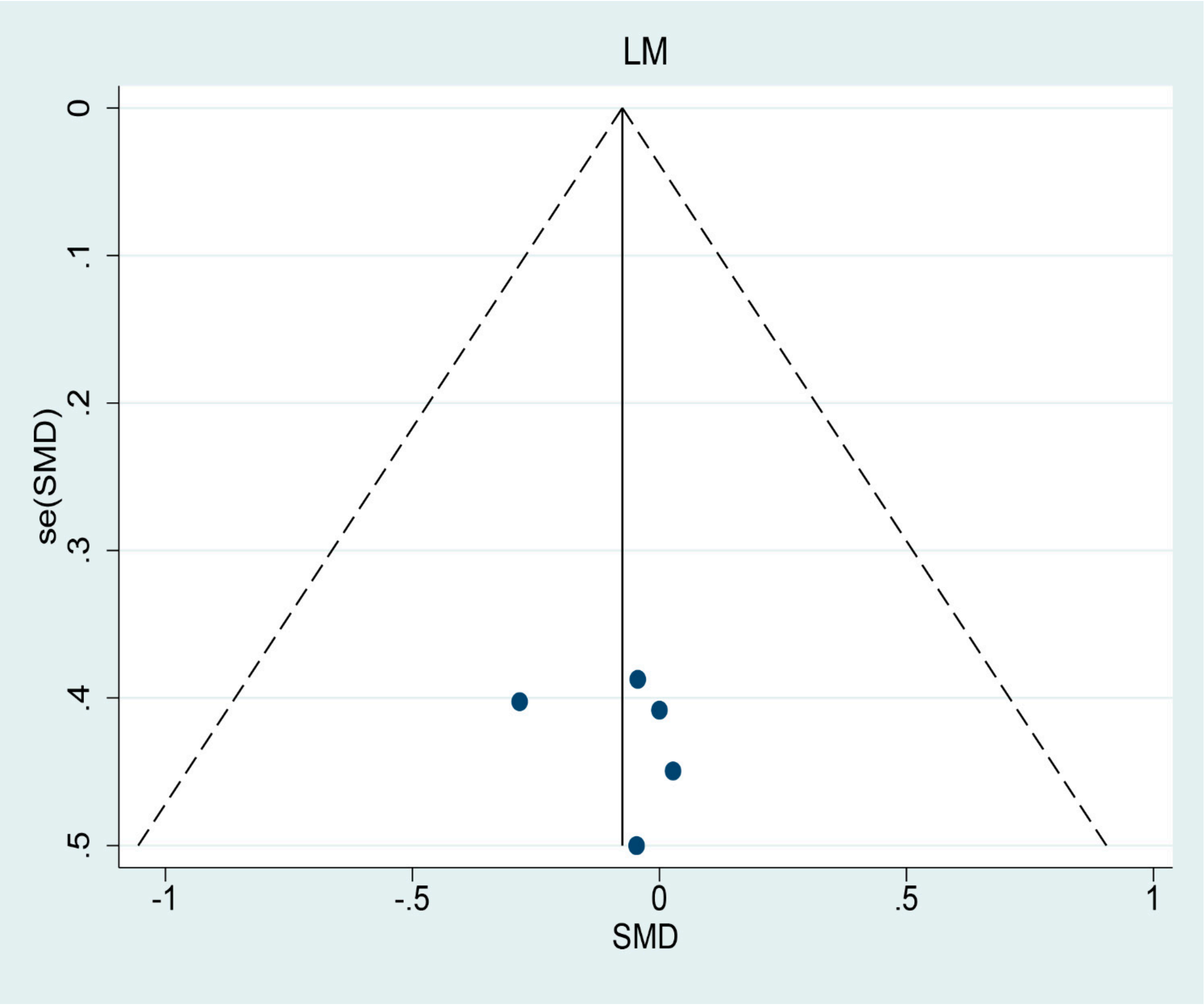

Supplementary Figure S2A: Forest plot of the effect of CT combined with LCHF on body fat percentage in recreationally trained or professionally trained participants.

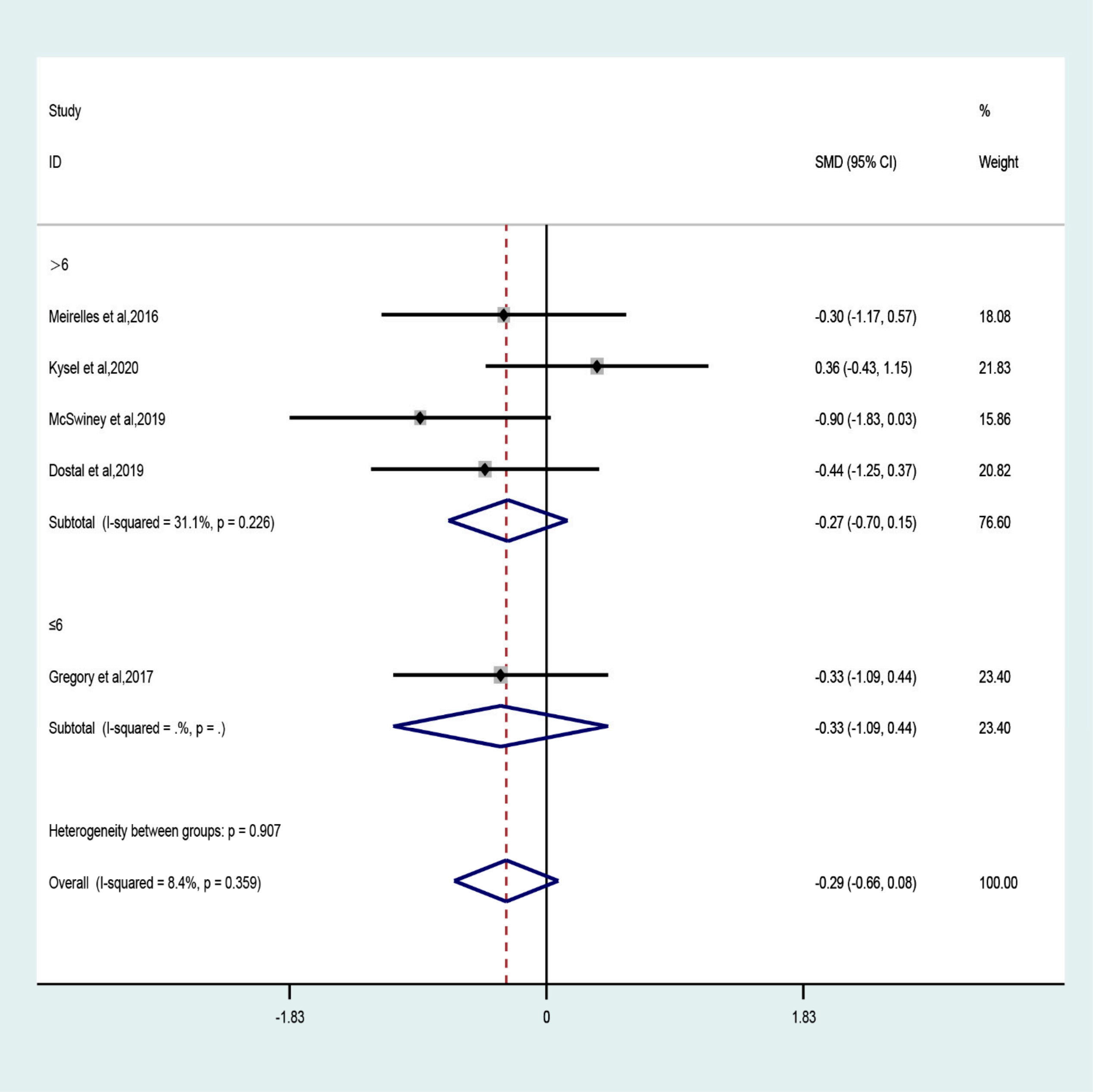

Supplementary Figure S2B: Forest plot of the effect of intervention duration (≤6 weeks or ＞6 weeks) on body fat percentage.

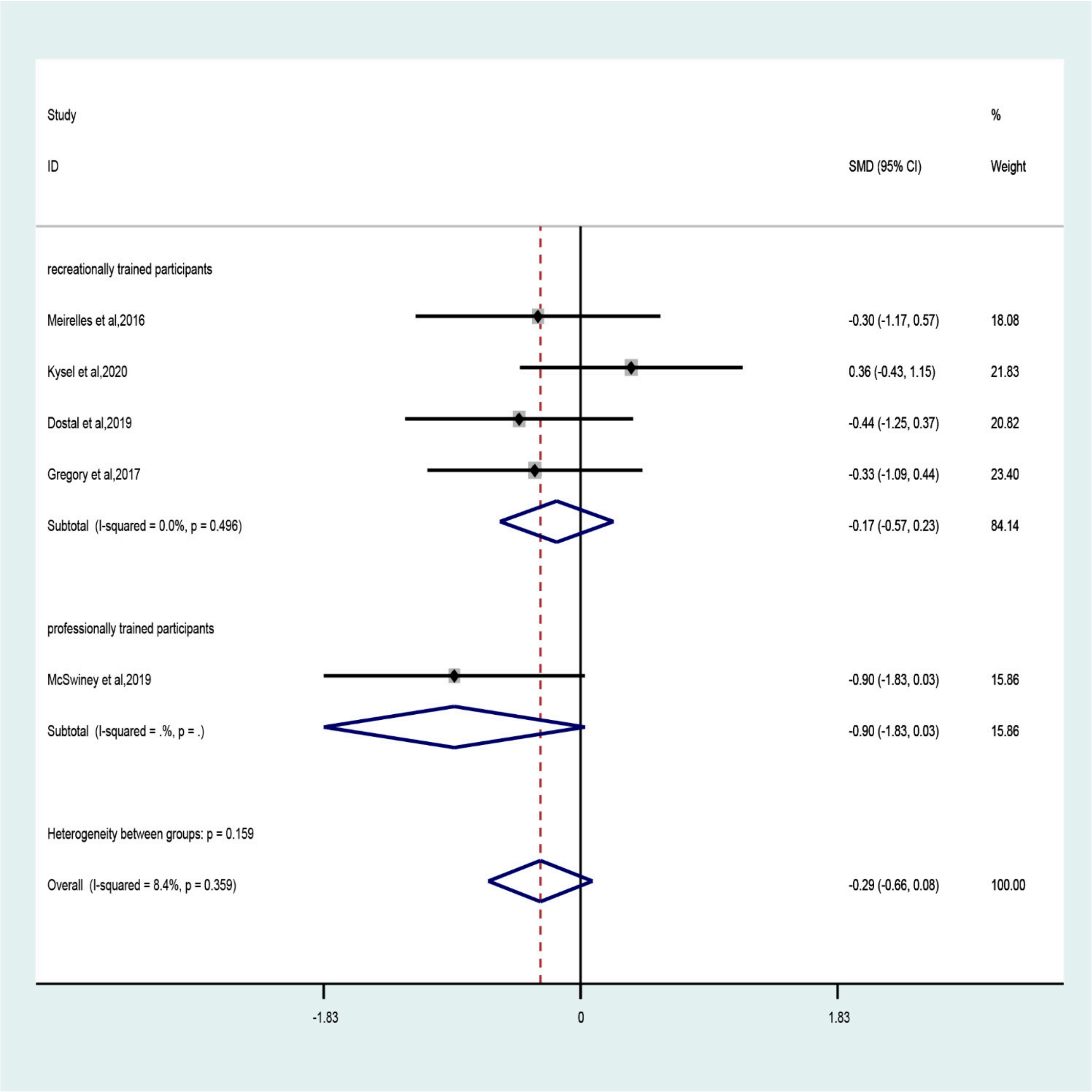

Supplementary Figure S3: Funnel plot of the effect of CT combined with LCHF on body fat percentage to controls.

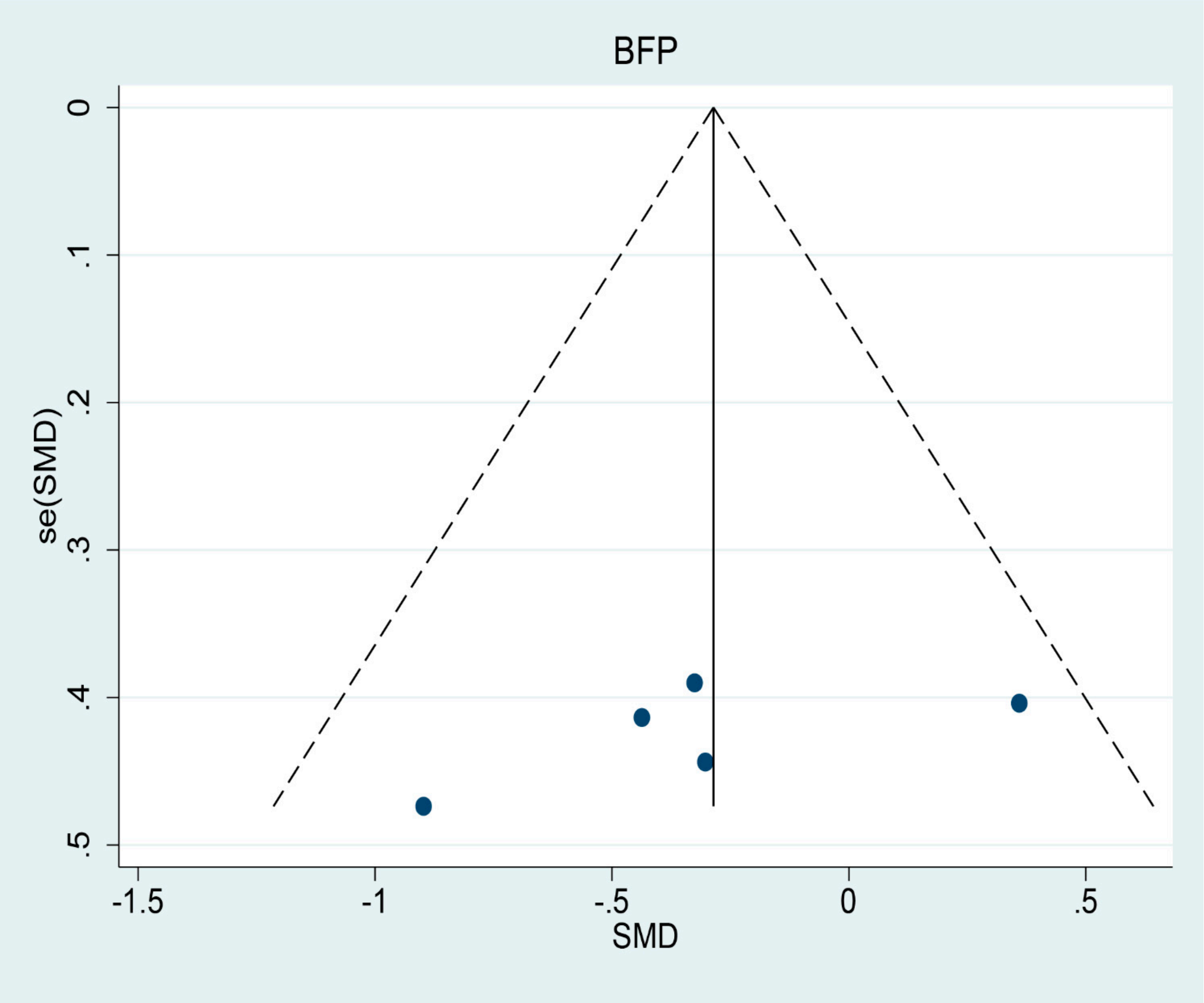

Supplementary Figure S4A: Forest plot of the effect of CT combined with LCHF on body mass in recreationally trained or professionally trained participants.

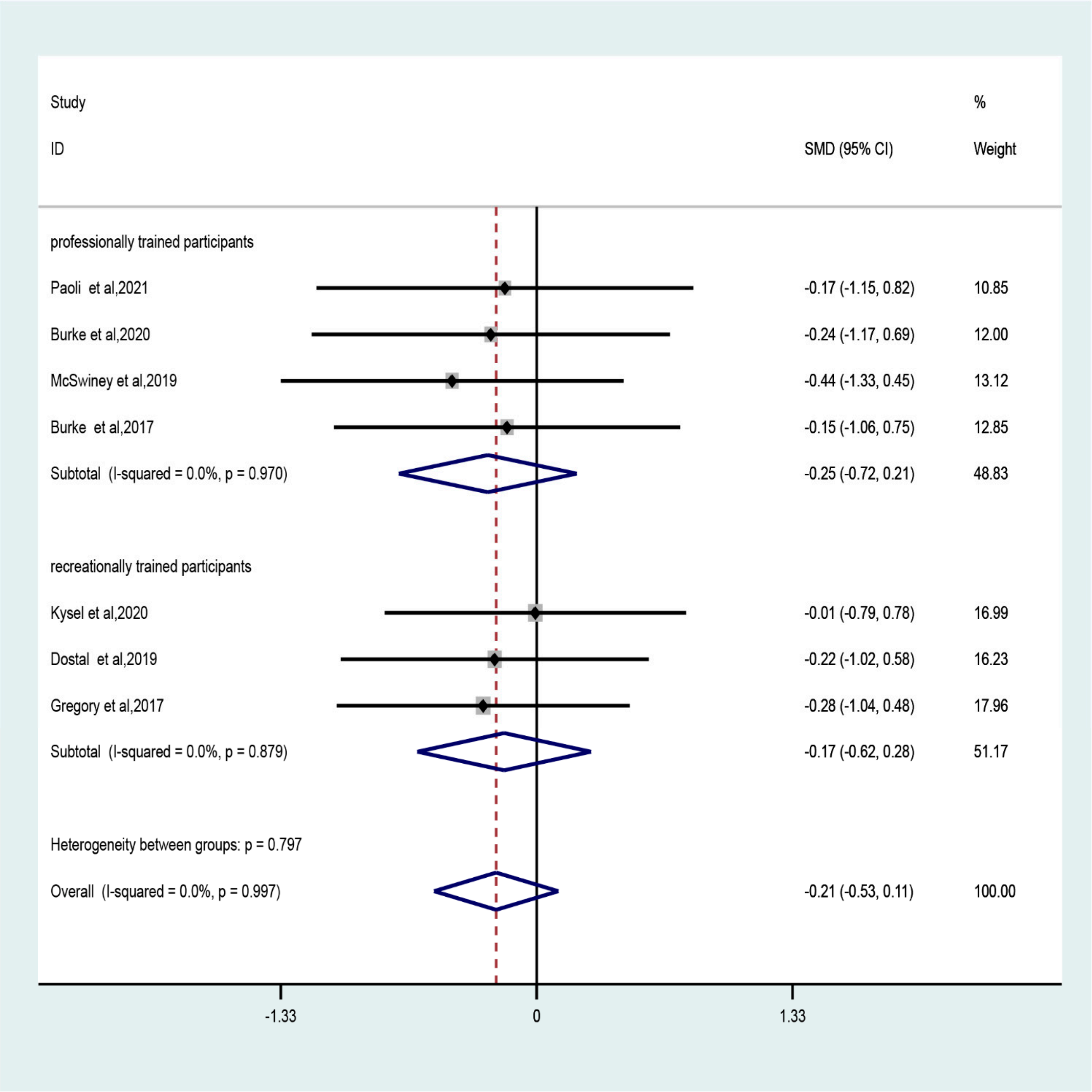

Supplementary Figure S4B: Forest plot of the effect of intervention duration (≤6 weeks or ＞6 weeks) on body mass.

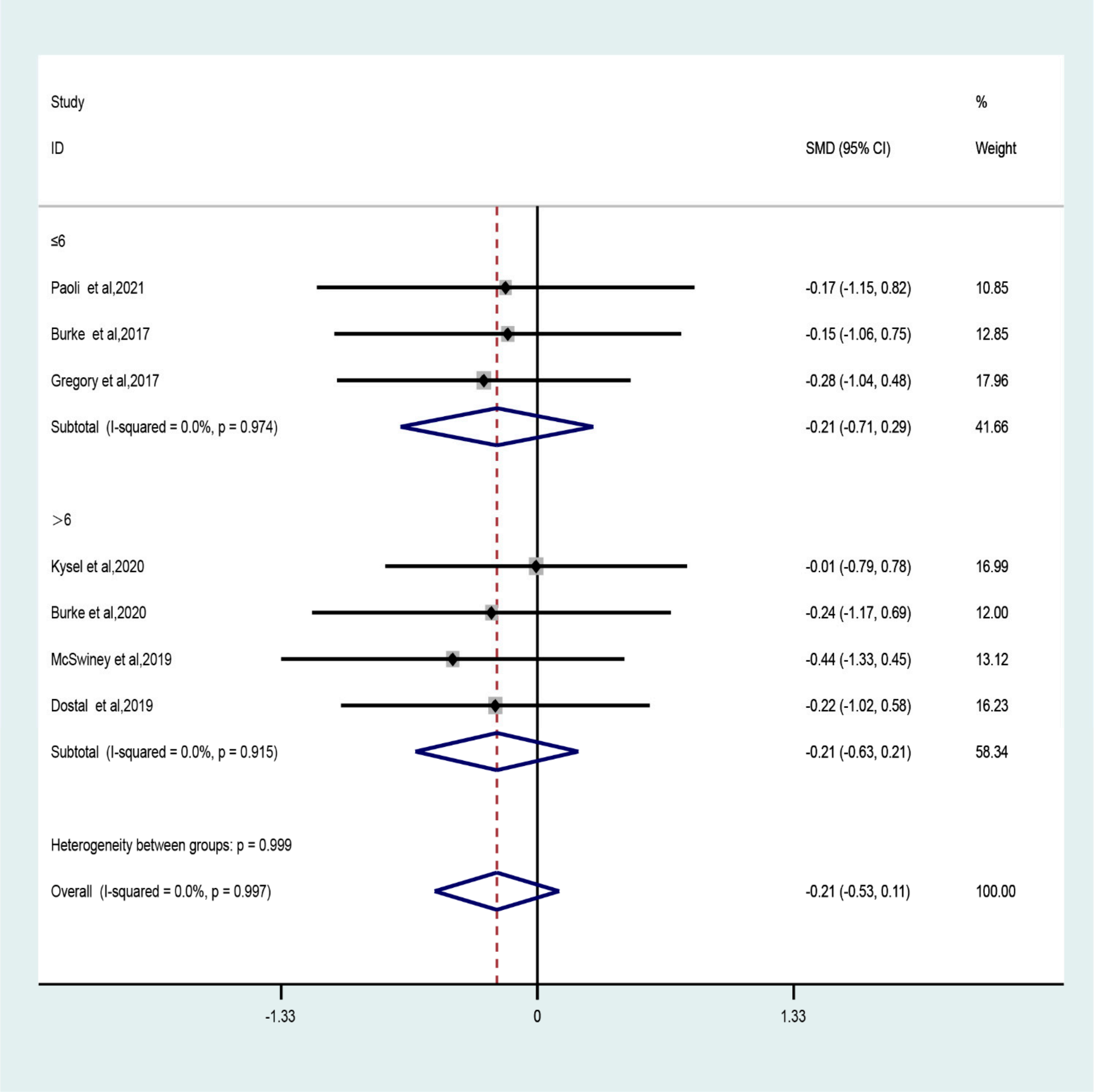

Supplementary Figure S5: Funnel plot of the effect of CT combined with LCHF on body mass.

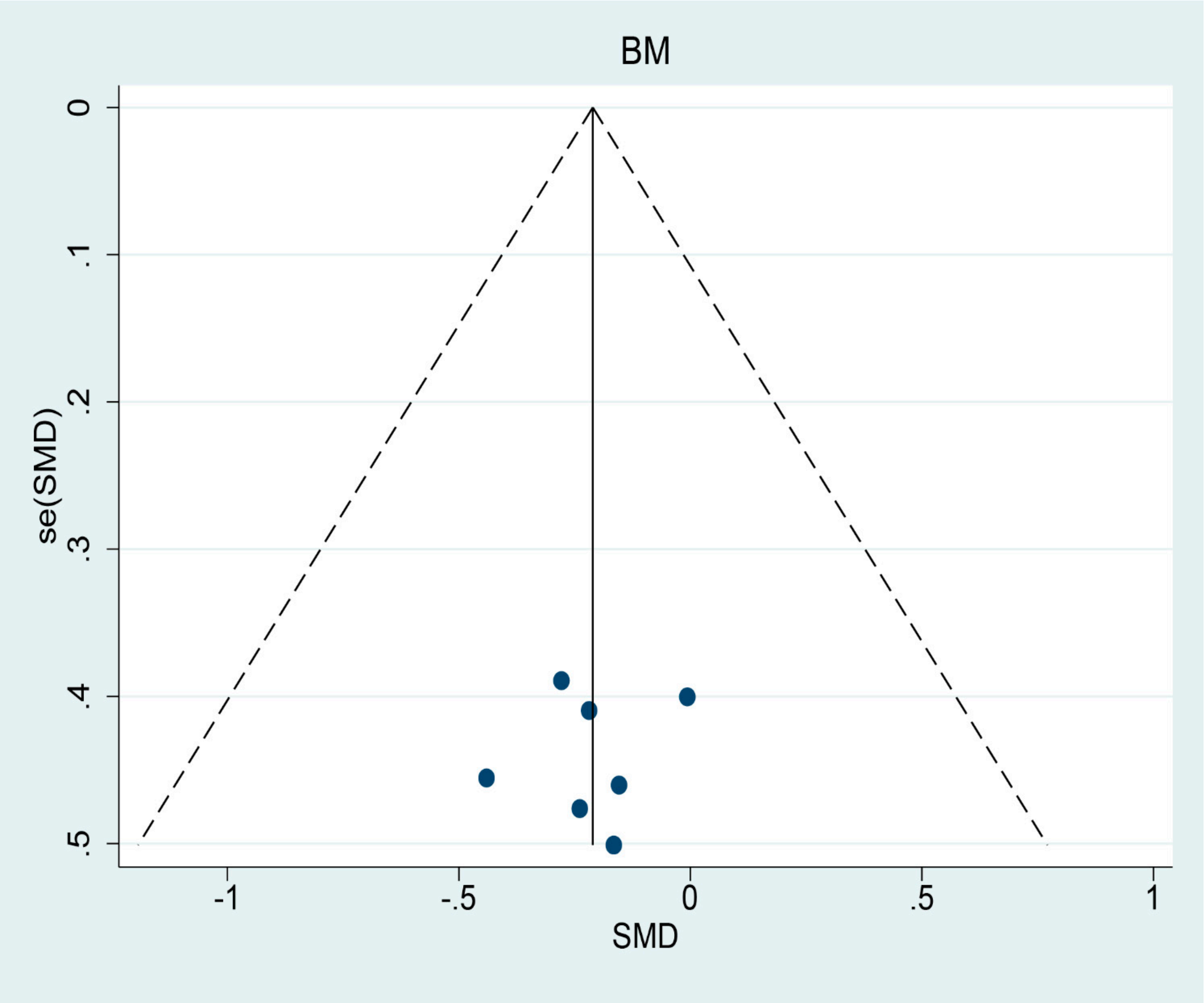

Supplementary Figure S6: Funnel plot of the effect of CT combined with LCHF on VO_{2max}.

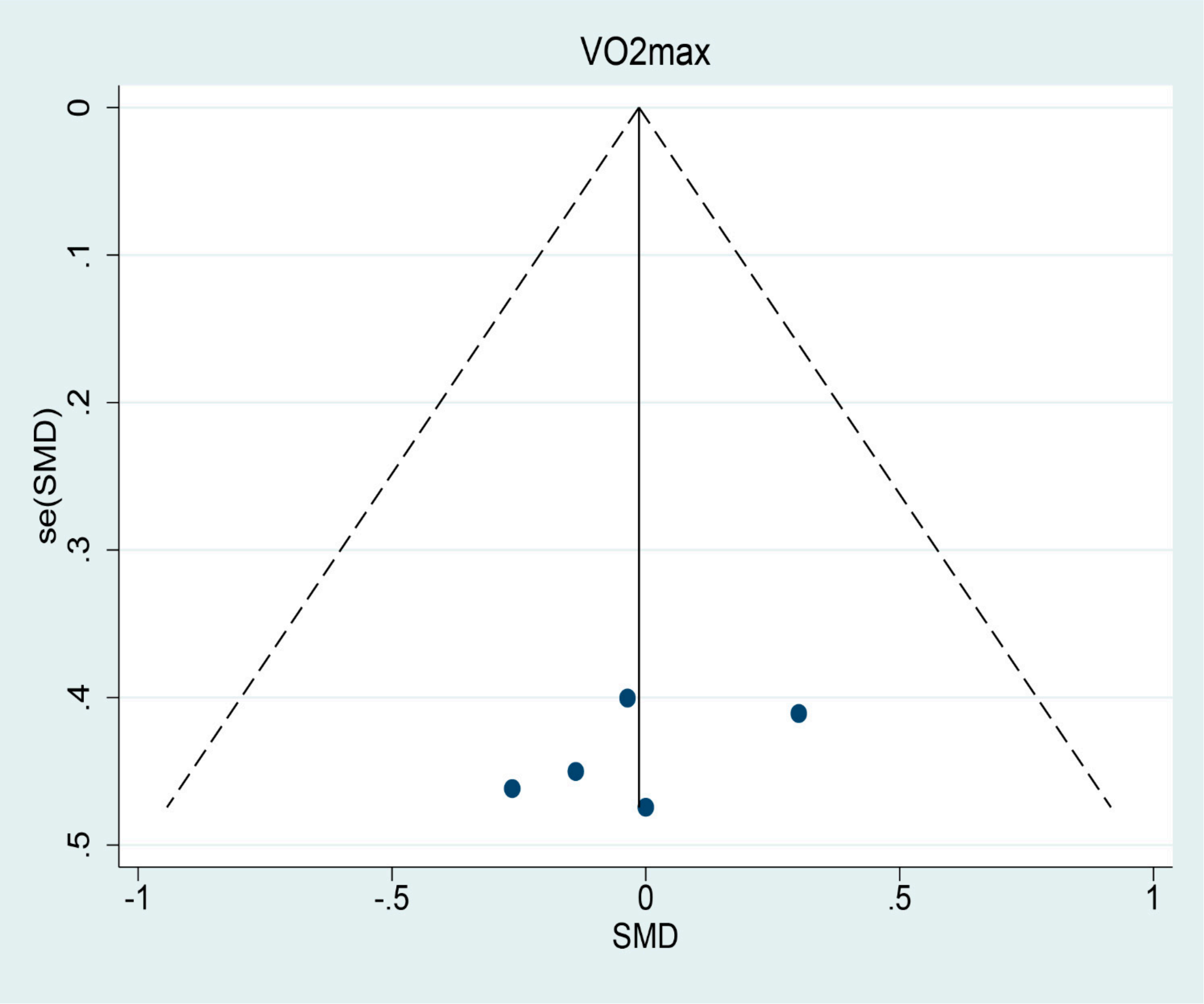

Supplementary Figure S7A: Forest plot of the effect of CT combined with LCHF on aerobic performance in recreationally trained or professionally trained participants.

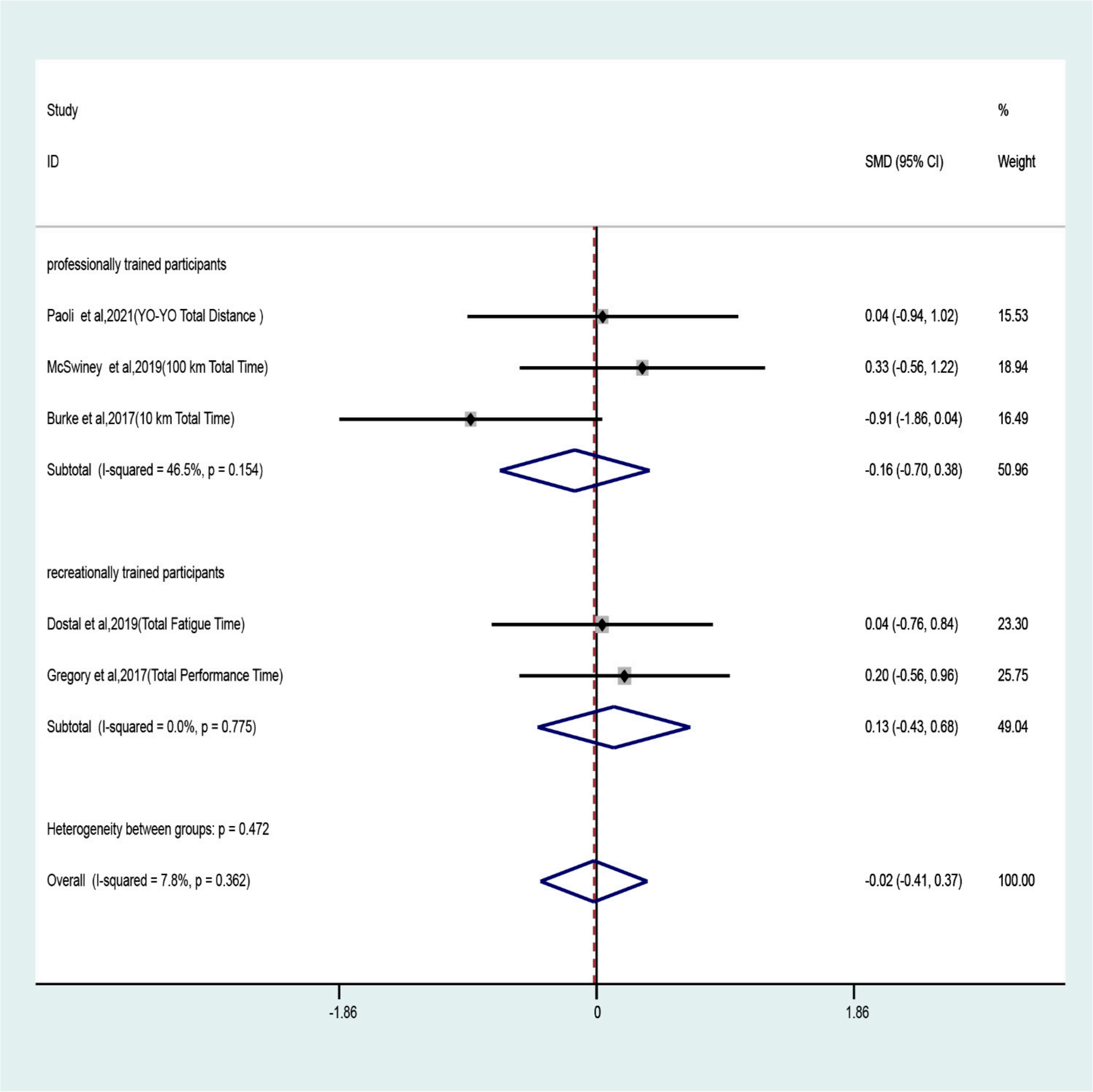

Supplementary Figure S7B: Forest plot of the effect of intervention duration (≤6 weeks or ＞6 weeks) on aerobic performance.

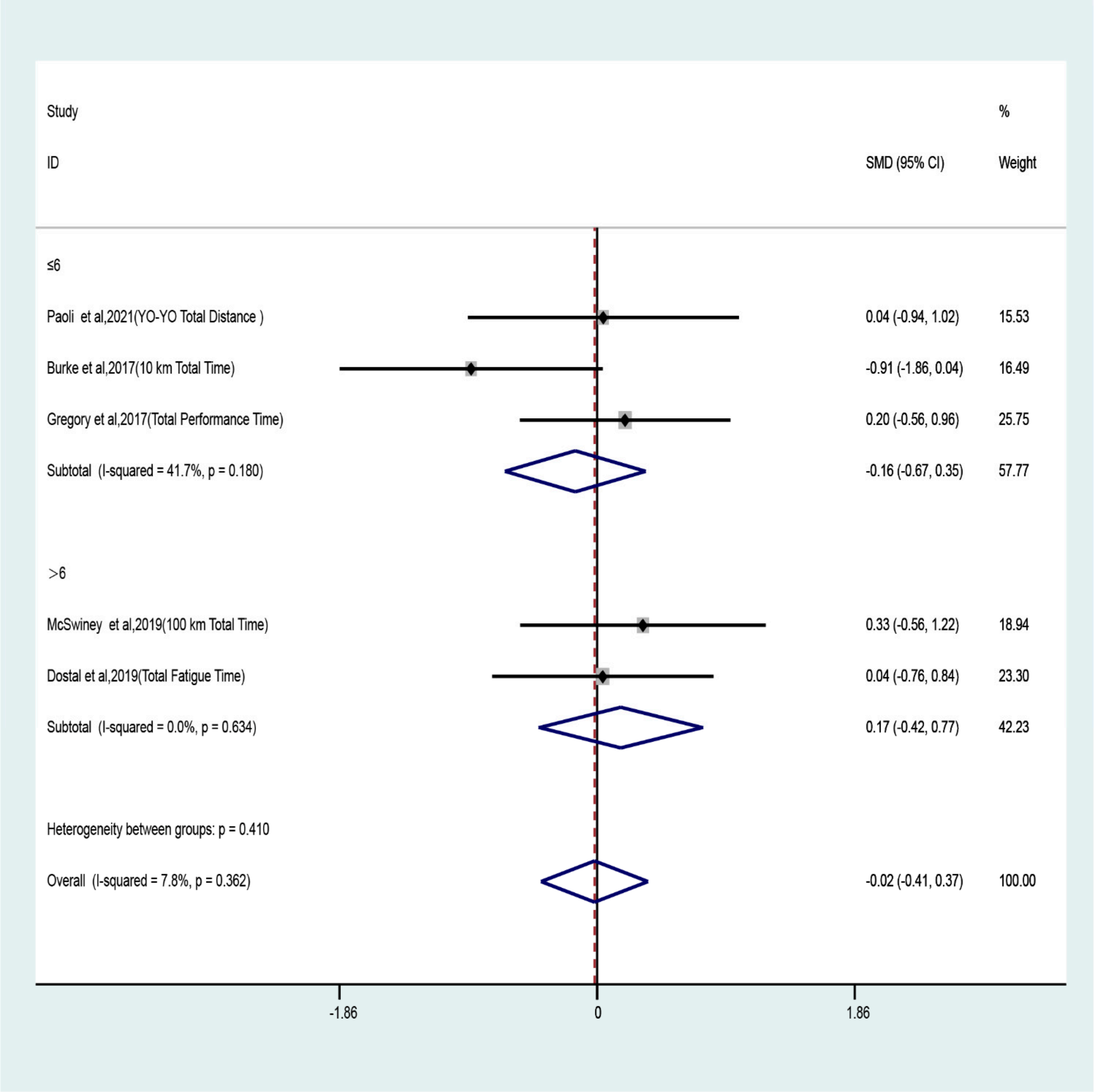

Supplementary Figure S8: Funnel plot of the effect of CT combined with LCHF on time (or distance) to complete the aerobic tests.

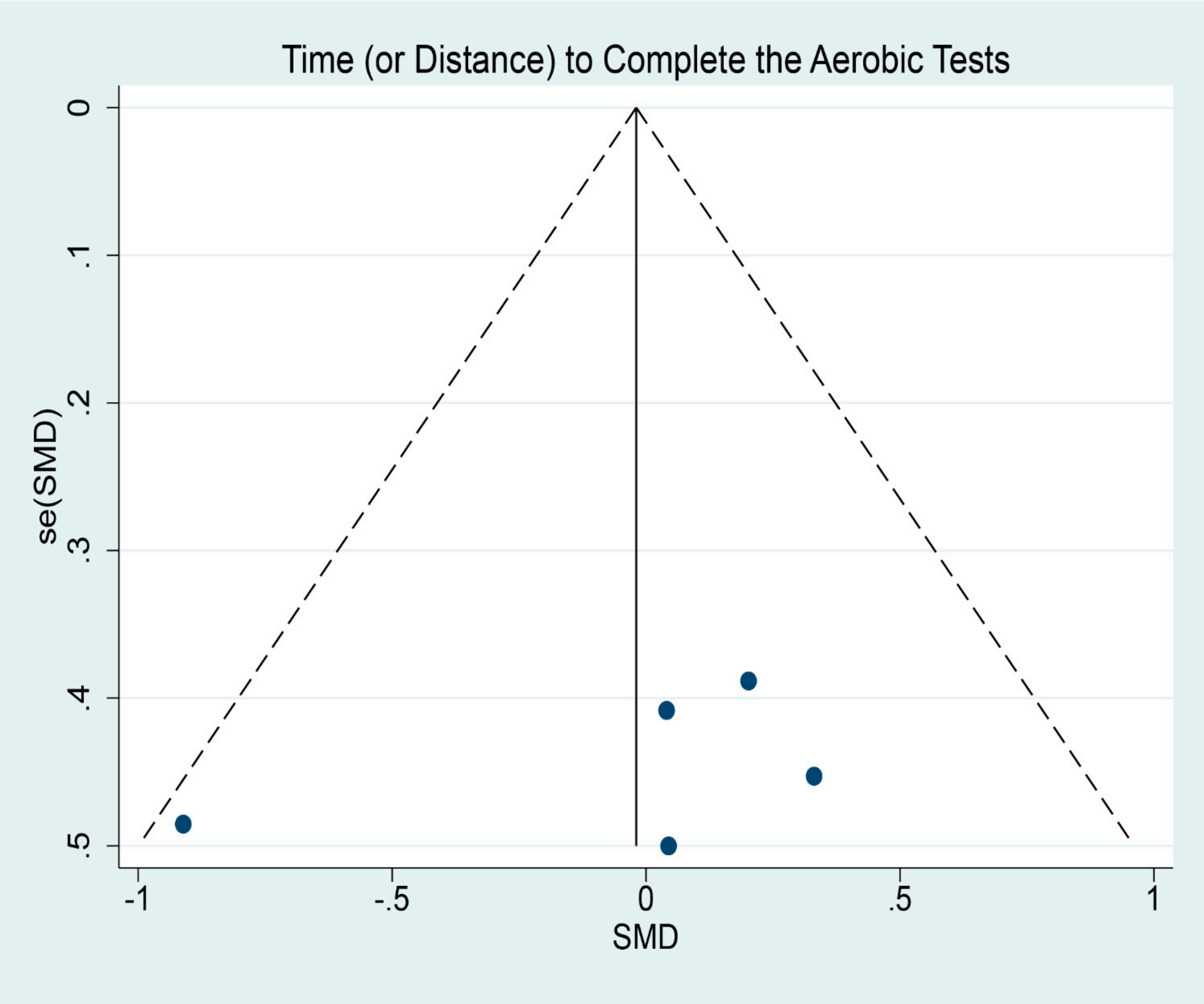

Supplement: Supplementary file 1 [file ijerph-19-11542-s001.zip › ijerph-1890043-SI.pdf]
